# A New Method of Applying the Tourniquet, to Restrain Arterial Hemorrhages from the Lower Extremities

**Published:** 1799-03

**Authors:** A. Carlisle

**Affiliations:** Surgeon to the Westminster Hospital


### *A New method of applying the Tourniquet, to reftrain Arterial Hemorrhages from the lower extremities: by* A. Carlisle, *Surgeon to the Weftminfter Hofpital.*

In the practice of many chirurgical operations, and alfo in a variety of cafes which occur through accidents or difeafes, it frequently becomes an object of great importance, to fupprefs the bleeding from arteries, without intercepting the circulation throughout the whole limb. In fuch cafes, the following method of applying a tourniquet to the lower extremities may be fafely recommended to the practitioner. A hard roll of linen bandage, about four or five inches in width, and three in thicknefs, being provided, and a piece of fmooth board, nine inches in length, five in width, and three-quarters of an inch in thicknefs, with the fides and ends fquared at right angles, the roller is to be placed in the ham, mid-way between the external and internal flexor tendons on the under fides of the knee joint, the leg being extended in a ftraight line ; the piece of board is then to be placed over the roller, which is to act as a pad of compreffion on the popliteal artery, the length of the board running crofs-ways and projecting beyond the knee-joint on each fide.

The

The girth of the tourniquet is to go round the knee above (not upon) the patella, and over the projecting ends of the board. The screw should rest at the upper part of the limb above the patella, having a pad interposed between it and the skin. This mode of compressing the popliteal artery is attended with an important advantage, it allows the arterial circulation by the lateral anastomising vessels to proceed uninterrupted; the large superficial veins also are undisturbed, so that the limb remains in the same state as if the artery alone had been tied.

It is obvious, that this mode of applying the tourniquet will not be proper at the time of amputation. In all cases however of hemorrhages, when there is a chance of saving the limb, it will be found preferable to the total stoppage of circulation by the ordinary methods. Also in gun-shot wounds, compound fractures, secondary hemorrhages after amputation below the knee, and in cases of necrosis, where portions of dead bone are to be removed by force, dangerous troublesome bleeding, from arteries enveloped in newly formed bone, where they cannot be secured by ligature, this mode of practice will prove advantageous.

*Soho Square, Feb. 2, 1799.*

