# Erratum

**Published:** 1799-04

**Authors:** 


——231. 23, for 'A new method of,' read *Proposals for applying, &c.*

